# Infantile Fibrous Hamartoma: A Rare Case of a Paraumbilical Subcutaneous Mass in a One-Year-Old Male

**DOI:** 10.1155/crpe/6588108

**Published:** 2025-11-21

**Authors:** Fahd Refai, Shahad Isa

**Affiliations:** Department of Pathology, Faculty of Medicine, King Abdulaziz University, Jeddah, Saudi Arabia

## Abstract

Infantile fibrous hamartoma (FHI) is a benign soft tissue tumor that primarily occurs in infants. We present the case of a one-year-old male with a paraumbilical subcutaneous mass. Clinical differentials included lipoma versus paraumbilical hernia. Ultrasound revealed a hypoechoic, elongated, poorly defined nodule in the right upper anterior abdominal wall, while immunohistochemistry showed positivity for BCL2, smooth muscle actin (SMA), and cytoplasmic *β*-catenin. Surgical excision and pathological examination revealed an ill-defined lesion composed of abundant mature adipose tissue interspersed with densely hyalinized fibrous tissue. Paraumbilical FHI can clinically mimic a lipoma or a small hernia. Correlation with targeted ultrasound assists in excluding hernia and guiding surgical management. Surgical removal is generally curative, with a low recurrence rate. This case contributes to the literature on FHI and underscores the importance of considering this entity in the differential diagnosis of subcutaneous masses in infants.

## 1. Introduction

Fibrous hamartoma in infants was first described by Reye in 1956 [1] and later termed “fibrous hamartoma of infancy” (FHI) by Enzinger in 1965 [2]. FHI is a soft tissue mass with triphasic histopathology consisting of fibromatosis, myxoid adipose tissue, and mesenchymal tissue [3]. It is rare and usually affects children under 2 years of age, although misdiagnosis is common [3, 4]. Congenital FHI occurs in ∼15% of children, and 90% of cases are diagnosed within the first year of life [3, 5]. Approximately 300 cases have been reported to date [6]. The condition has a male predominance and most often affects the scrotum, sacral region, trunk, or limbs [7]. Clinically, FHI usually presents as a painless mass but can also appear with multiple nodules, skin discoloration, edema, hypertrichosis, or skin tethering [8]. Lesions typically grow rapidly but remain asymptomatic, have a favorable prognosis, and rarely undergo sarcomatous transformation [6]. Because FHI closely resembles malignant sarcomas radiologically and clinically, excision is essential both for diagnosis and treatment, with recurrence rates of < 15% [9]. Herein, we report the diagnosis and management of an asymptomatic one-year-old male infant with FHI.

FHI is uncommon in early childhood. Paraumbilical/umbilical involvement has been reported [10], and an axillary case has been described [11].

## 2. Case Presentation

A one-year-old male with no significant medical history presented with a 6-month history of a paraumbilical subcutaneous mass. The solitary swelling measured 3 × 2 cm, was mobile, and not attached to underlying structures. It was nontender, and the child exhibited no systemic symptoms such as fever or weight loss. Preoperative differential diagnosis was lipoma versus paraumbilical hernia.

Targeted ultrasound was requested to assess for a fascial defect and herniated contents. Localization of the lesion to the subcutaneous plane superficial to the rectus, absence of internal vascularity on color Doppler, and the explicit lack of a hernia sac or bowel/omental protrusion effectively excluded hernia and supported proceeding to excision. Ultrasound revealed “a 0.6 × 2.0 × 2.0 cm hypoechoic, elongated, poorly defined subcutaneous nodule in the upper anterior abdominal wall, superficial to the rectus muscle, corresponding to the area of clinical swelling in the paraumbilical region. The lesion shows a small central anechoic component and no internal vascularity on color Doppler. No evidence of hernia is identified.”

**Figure 1:**
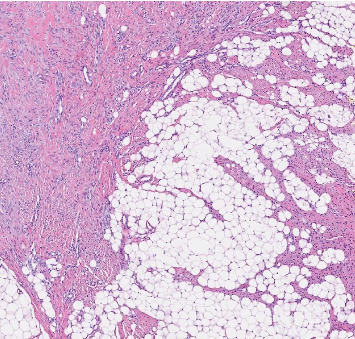
H&E stain, × 200 magnification, showing hyalinized fibrous tissue with slit-like spaces lined by bland spindle cells.

The patient underwent surgical excision. Histopathology revealed an ill-defined lesion within the subcutaneous tissue composed of mature adipose tissue interspersed with densely hyalinized fibrous tissue containing slit-like spaces lined by bland spindle cells. No hyperchromatic, multinucleated, or floret-like giant cells were observed, and mitotic activity was minimal. No necrosis or calcification was present (Figure 1).

**Figure 2:**
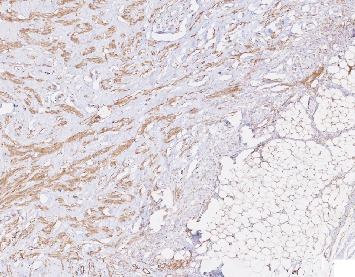
Immunohistochemical staining for BCL2, × 200 magnification, demonstrating positive cytoplasmic staining in spindle cells within the lesion.

**Figure 3:**
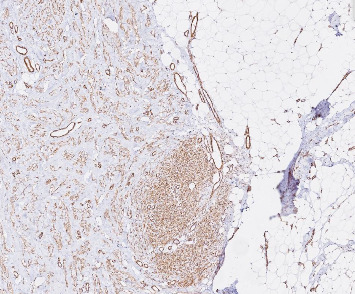
Immunohistochemical staining for smooth muscle actin (SMA), × 200 magnification, showing positive staining in spindle cells consistent with myofibroblastic differentiation.

**Figure 4:**
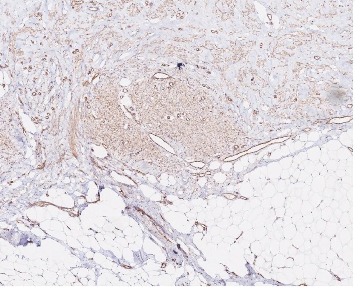
Immunohistochemical staining for *β*-catenin, × 200 magnification, displaying cytoplasmic expression in spindle cells, characteristic of fibrous hamartoma.

Immunohistochemistry showed positive staining for BCL2, smooth muscle actin (SMA), and cytoplasmic *β*-catenin (Figures 2, 3, 4). These findings supported the diagnosis of infantile fibrous hamartoma.

## 3. Discussion

On ultrasound, lipoma is usually well defined and predominantly echogenic (fatty) with thin internal echoes [12], whereas a paraumbilical hernia requires demonstration of a fascial defect with protrusion of omentum or bowel and dynamic change with Valsalva/crying [13]. In this case, the lesion was confined to the subcutaneous plane superficial to the rectus, with no fascial defect or herniated contents; these features favored a nonhernia soft-tissue lesion such as FHI, with definitive confirmation by histopathology [14]. Clinically, in infants with a small, soft paraumbilical swelling, the principal differentials are lipoma and paraumbilical hernia; lack of fluctuation with straining and absence of a palpable fascial defect support a nonhernia process [13].

FHI is rare, with ∼20% of cases present at birth and most diagnosed before 2 years of age, with a mean age of 15 months [8, 10]. Clinical presentation is usually a firm, asymptomatic subcutaneous mass without epidermal changes [10], consistent with our case.

Previous case reports describe similar findings in various regions, including the umbilical area [10], axilla [11], iliac region [15], leg [16], and scrotum [17]. An overview of previous cases is given in Table 1, summarizing the key findings and details from each report.

Imaging studies, particularly MRI, typically show fat interspersed with fibrous bands [14, 18, 19]. Our case was diagnosed by ultrasound, which remains a cost-effective tool for evaluating pediatric soft tissue masses.

Surgical excision is curative in most cases [6, 10]. In the largest review, Al-Ibraheemi et al. studied 145 cases and found common sites to include the axilla, back, upper arm, scrotum, chest wall, and thigh [6].

Histopathologically, FHI demonstrates three main components: myofibroblastic tissue, mesenchymal cells, and adipose tissue [10]. Our findings of mature adipose tissue interspersed with hyalinized fibrous tissue fit this pattern. Immunohistochemistry is useful for differentiating FHI from other pediatric soft tissue tumors [7, 20]. Typically, fibrous tissue is positive for vimentin and SMA, adipose tissue for S100, and mesenchymal tissue variably for actin and desmin [6, 10]. Our case showed positivity for BCL2, SMA, and cytoplasmic *β*-catenin. Interestingly, Yu et al. reported negativity for BCL2 and *β*-catenin [3], underscoring the heterogeneity of this entity.

Less than 300 cases have been documented [6]. Although most demonstrate triphasic morphology, diagnosis can be challenging due to variability in clinical and immunohistochemical presentation [19]. FHI has been reported in diverse anatomical sites, but paraumbilical presentation is extremely rare, with only one similar report to date [11].

Limitations of this case include its retrospective design, lack of long-term follow-up, and inherent challenges of case reporting, such as publication bias [21, 22].

## 4. Conclusion

This report highlights infantile fibrous hamartoma presenting as a paraumbilical subcutaneous mass that clinically mimicked lipoma or a small hernia. Targeted ultrasound aided in excluding a fascial defect, and surgical excision with histopathology established the diagnosis.

## Figures and Tables

**Table 1:** Previous case reports of infantile fibrous hamartoma.

Case (reference)	Age/sex	Clinical presentation and site	Imaging/diagnostics	Pathology/IHC findings	Management	Outcome
Muñoz et al. [10]	Newborn, male	Erythematous plaque, periumbilical	US: hypoechoic dermoepidermal lesion; biopsy: biphasic fusiform cell morphology	IHC: CD34+, FXIIIa+	Surgical excision	No recurrence at 7 months
Demirtaş-Güner et al. [11]	15 months, male	Painless axillary mass	US: heterogeneous nodular lesion, mixed echogenicity, poorly defined margins	Histology: consistent with FHI	Surgical excision	No recurrence at 3 years
Farho et al. [15]	11 years, male	Painless iliac region mass	US: adipose-like echogenicity, mild perfusion on Doppler	Triphasic pattern; IHC: CD34+	Surgical removal	Asymptomatic, 7-month follow-up
Lama et al. [16]	18 months, female	Swelling, proximal right leg	US: heterogeneous soft tissue mass; FNAC: spindle cell lesion	Histology: triphasic; Alcian blue+	Surgical excision	Follow-up duration not reported
Kim et al. [17]	5 months, male	Painless right inguinal/scrotal mass	US: ill-defined fusiform mass; CT: spongiform solid lesion with heterogeneous enhancement	Histology: triphasic; IHC: CD34+, S100+	Surgical excision	Follow-up duration not reported
